# Methods of Controlling Hemorrhage of the Oral Cavity

**Published:** 1903-07

**Authors:** H. E. Belden


### METHODS OF CONTROLLING HEMORRHAGE OF THE ORAL CAVITY.[1]

BY H. E. BELDEN, M.D.

I HAVE been asked by the secretary on Stomatology to read a paper which would be of interest to the medical as well as to the

[1] Abstract of a paper read before the American Medical Association, Section on Stomatology, New Orleans, May 5 to 8, 1903.

dental profession. I have selected for my subject, "Methods of controlling Hemorrhage of the Oral Cavity." This may not be as important to the general practitioner as to the specialist; still, as the blood is the life-fluid of the body, any suggestions to prevent its loss will, I think, be of interest to the profession at large, especially as the pathology and treatment of hemorrhage of the oral cavity do not differ materially from that of hemorrhage generally.

The pathology of the hemorrhagic diathesis is not perfectly understood, and its actual cause cannot always be satisfactorily explained. It is quite evident that the bleeding continues from a lack of harmony between the coagulation of the blood and the contractility of the vessels.

There is a tube invented by Wright, by which the coagulation time of the blood can be determined. The blood is sucked into the tube and its fluidity tested at intervals of less than a minute.

The normal time for blood coagulation is from two to four minutes, but in the hemorrhagic diathesis, and in certain diseases, the time is so extended that a clot does not form quickly enough to engage the fibrin and act as a plug in the mouth of the vessels, but, on the contrary, coagulating in from six to twenty minutes, the clot, forming outside, acts as a vehicle to hold the wounded surfaces apart and facilitates the free oozing of the blood. Therefore hemorrhage continues from a perverted relation between the constituents of the blood which are necessary to form a clot (fibrin ferment, fibrinogen, and paraglobulin) and the contractility of the circular muscular fibres of the arteries, veins, and capillaries.

We know that the vasomotor system of nerves not only acts on the entire vascular system as a whole, but can control the blood to a circumscribed locality. So in the wounds caused by leeches, bleeding continues for hours after the removal of the leech. Could this not be explained by the fact that the leech has produced a circumscribed vasomotor paralysis?

I think that an important point in the arrest of hemorrhage is that the doctor impress the mind of the patient with his ability to control the hemorrhage, thus overcoming the effect that fear has on the vasomotor centres as they control the contractility of the muscular coat of the vessels.

I have noticed, after the extraction of teeth, that fear has a tendency to increase the hemorrhage. For example, Miss T. came into my office the other day to have a tooth extracted. She is not of the hemorrhagic diathesis, but presented a marked case of surgical fright. After the extraction she bled profusely.

Dr. J. A. Bodine, in a recent clinical lecture, cites a number of cases where death has been caused by fright. He says that the autopsy findings are exactly the same in cases of death from fright as in those from chloroform poisoning, the cause of death in both cases being a vasomotor paralysis, "that is to say, the nervous system lost control over the motor nerve leading to the blood-vessels, and especially the veins of the body, and as a consequence the patient bled to death into his own tissue."

The simple term hemorrhage is used when the blood passes outside the body.

If it escapes *within* the cavities of the body it is known as *internal* hemorrhage. When a small quantity of blood is forced or infiltrated into the tissues, extravasation is produced.

Internal hemorrhage may occur in two different ways,—by rupture of the vessels, and by diapedesis. In diapedesis the corpuscles of the blood escape into the tissues through the walls of the vessels.

The control of hemorrhage depends upon its cause. The cause may be accidental, that is, come from injury or disease, or it may be intentional, as in operations or the extracting of teeth.

The hemorrhage may be arterial, venous, or capillary, but is often a combination.

When there is injury to the arteries or large veins, ligature is necessary, and when to the smaller veins and capillaries, we resort to sutures, compression, and styptics.

After the extraction of teeth prolonged bleeding might occur from the dental artery becoming entangled in the ragged edges of the alveolus and its mouth being held open. In this case the bleeding would be red blood and spurt in jets. A drill run down into the cavity would disentangle the artery and stop the bleeding. In the same way it is possible that a nerve might become entangled. This would cause neuralgia. Relief would respond to the same treatment.

With proper treatment, death is seldom a result of hemorrhage of the oral cavity. When it does occur it generally comes from the hemorrhagic diathesis or from accidental puncture to the larger vessels, as the jugular vein and carotid artery, or, in cases of cancer and other malignant growths, to capillary bleeding from the slough of unhealthy granulations. When the hemorrhage occurs from this cause the general condition of the patient is so serious that death is imminent anyway.

The treatment of hemorrhage of the oral cavity may be classed under three heads,—mechanical, medicinal, and physiological.

The mechanical methods consist of direct application, as plugging or tamponing, compression, ligating of the artery, tortion and suturing, position of the patient, etc.

As hemorrhage of the oral cavity is usually capillary, compression is often all that is necessary to control it, when properly applied.

In this connection, I will tell of the heroic treatment resorted to with good results by Dr. Fry, of Hebron, Neb., of which I lately read in the *Dental Digest.* Tooth extraction was followed by hemorrhage in a patient subject to bleeding. He took an impression of the mouth in modelling compound; when cold, he trimmed it to make an appliance that could be worn in the mouth, notching it to receive a strong bandage to be tied over the head. He then made a thin batter of plaster, added a pinch of salt, and filled the appliance, putting it at once into the mouth and pressing it into place. When the plaster was set he added the bandage and tied it firmly. The socket had previously been packed with fluid extract of ergot. Immediate relief was given.

In cases where a predisposition to hemorrhage is known or suspected, before operating it is well to give systemic treatment, the treatment depending upon the general condition of the patient.

Among the new remedies which have appeared is calcium chloride. The *Medical Brief* has this to say of it: "Calcium chloride, in doses of from eight to sixteen grains every two to four hours, should be tried in all forms of persistent hemorrhage. This salt increases the coagulability of the blood, but if used more than three days consecutively, it has the opposite effect."

In a recent number of the *Dental Cosmos* is the following, taken from the *Revue de Stomologie,* of Paris:

"Use of Calcium Chloride to prevent Hemorrhage after Tooth Extraction.—As is well known, the extraction of a tooth may give rise to severe hemorrhage in persons suffering from hæmophilia. Dr. C. E. Vallis, assistant dental surgeon in King's College Hospital, London, has observed the case of a woman, aged twenty-five years, presenting the hemorrhagic diathesis, in whom the extraction of a tooth was followed by a hemorrhage which lasted thirty-six hours. As the teeth of this patient were in very bad condition, and as the extraction of all the carious teeth became necessary because of dyspeptic troubles from which the patient was suffering, Dr. Vallis endeavored to use some means by which the coagulability of the blood would be increased. With that object in view he administered calcium chloride in weak doses during a period of eight days previous to the time set for the performance of the operation. He extracted an incisor, an operation that was performed without the slightest loss of blood. Continuing to administer the same agent, he was able to extract every tooth without hemorrhage. Since then Dr. Vallis has observed a similar case in which calcium chloride has given the same satisfactory results. The disagreeable taste of this medicament, and the slight tendency to constipation which it induces, are the only inconveniences connected with its administration, even after a continuous use during a period of three to four weeks."

The *Medical Journal* gives the following formula ascribed to Bertignon:

| | | |
|---|---|---|
| Crystalized calcium chloride . . . . . . | 4 grammes | 60 grains. |
| Syrup of mint . . . . . . . . . . . . . | 30 grammes | 1 ounce. |
| Distilled water . . . . . . . . . . . . . | 90 grammes | 3 ounces. |

To be taken in the twenty-four hours, a tablespoonful every two hours.

Dr. A. J. Ochsner gives a method of treatment in cases of hæmophilia, which consists of giving albumin in the form of whites of eggs. He says, " When from four to six whites of eggs are given three times daily there seems to be a very definite effect exerted upon the clotting properties of the blood of these persons."

Another remedy which increases the coagulability of the blood and is creating general attention as a hæmostatic is *gelatin.* It should be given in large doses, from one hundred to three hundred grains daily, and can be continued indefinitely. It seems to be contraindicated only in Bright's disease. It is doubtful whether it should be used subcutaneously or intravenously, as cases of tetanus have been reported from its use. We can understand this, for we know that gelatin is the medium used for propagating bacteria; and several authorities give it as their opinion that sterilization destroys its hæmostatic properties. When applied locally, an antiseptic should always be used in conjunction with it. It is a splendid systemic remedy, and the good results consequent upon its use are probably due to the nourishment of the vasomotor centres.

The following I have taken from the *New York Medical Journal,* copied from the *Progrès Médical* of April 5, 1902: " M. Marc Laffont and M. André Lombard conclude that whenever some nutritional vice modifies the cryoscopic and other properties of the blood there may be a glycosuria, an albuminuria, or a hemorrhage. Whether this syndrome is accompanied or not by an anatomical lesion, in a great majority of instances the lesion is curable if one attacks the cause. Gelatin seems to be the only agent capable of rendering the plasticity of the blood normal, when administered in the dose of two hundred and twenty-five grains daily. It is a harmless drug and no contraindication to its use exists, and its administration should be prolonged."

The *Philadelphia Medical Journal* has this to say of adrenalin: "Generally speaking, adrenalin, when locally applied, is the most powerful astringent and hæmostatic known; also a very strong stimulant of the heart. It is non-irritating, non-poisonous, and non-cumulative so far as it has been observed. It is indicated in a condition produced by morphine and opium poisoning. It has produced good results in circulatory failure, in prevention of collapse of anæsthesia, and allied conditions. It is invaluable in carrying out bloodless operations in nose, ear, eye, and throat work. Out of a great number of operations in which it was used, in only a few instances was sloughing after operation reported. The length of time required to control bleeding depends upon its strength."

The following points I have culled from the report of the drug in the last number of the *International Clinics.*

Adrenalin is six hundred and twenty-five times more active than the fresh suprarenal gland. The adrenalin chloride rapidly produces ischæmia of mucous surfaces, and is employed in oral surgery for performing bloodless operations.

Internally in large doses, it causes dyspnœa, lowering of the sensibility, diminished reflexes, and loss of voluntary movements. According to Tarasmio, death takes place by the paralysis of the central nervous system in frogs, and by pulmonary œdema in mammals. In rabbits 0.02 adrenalin per kilogram was always fatal when administered subcutaneously, but never by the use of 0.002 grains (grams).

I have lately been experimenting with adrenalin in a small way. I have used it with good result to arrest the gingival hemorrhage which sometimes occurs in the removal of tartar from the necks of teeth and to check the bleeding which comes from the abrasion of the gums caused by the slipping of an instrument, sand-paper disks, emory wheels, etc., also in the removal of epulic tumors and after the extirpation of the pulp of a tooth.

I have had good results consequent upon the use of adrenalin in conjunction with cocaine in tooth extraction. I take a dram of the solution (1 to 1000), add a grain of cocaine (which gives about a two per cent. cocaine solution), and inject a few minims into the gum. The cocaine produces the usual local anæsthesia, and the bleeding in almost every case has been extremely slight, and in some cases there has been no bleeding at all.

Bartholow says of turpentine: "It is a serviceable cardiac stimulant when the action of the heart is weak and the arterial tension low. In passive hemorrhages we possess few agents more generally useful. The indications for its use are a general debility, relaxation of the vessels, and an impoverished condition of the blood. It need hardly be stated that active hemorrhage or a condition of plethora contraindicates the use of turpentine."

According to Garretson, "Depressing the action of the heart is under almost all circumstances a valuable means to arrest hemorrhage. To this end veratrum viride is always given with satisfaction. The dose is five drops for an adult, given in a tablespoonful of water. Conjoined with this, and in many instances quite capable of taking its place, is the hot foot-bath."

Dr. Arthur Masur, of Breslau, Silesia, advises the use of powdered charcoal after tooth extraction where the hemorrhage requires special attention. Copied from the Review on Current Literature in the *Dental Cosmos,* his *modus operandi* is as follows: "The alveolus, which is freely irrigated with pure water, is dusted with charcoal powder, and, to insure better adaptation of the charcoal to the bleeding capillaries, it can be packed into the alveolus by means of cotton." There is also another and very easy way of carrying the powder into the alveolus,—by means of a damp cotton tampon, which is made to take up the charcoal and is then introduced into the canal. The powder is allowed to remain two minutes in the alveolus, and is then washed out with a strong stream of water.

In cases in which it is desirable to leave a packing overnight, the use of iodoform gauze impregnated with charcoal is recommended, although the essayist states that he has never had to recur to this procedure, as he has always been able to arrest hemorrhage in these cases with the simple application of charcoal powder.

The action of the charcoal powder is supposed to be merely a physical one, the closing up of the openings in the capillaries with the charcoal granules. Incidentally, it is also stated, the charcoal has the property of hastening the healing of the wound, and its union with the tissues of the wound is very intimate, as it takes a strong stream of water to dislodge it.

Dr. Spaark, in the *French Medical Journal*, advises a wash of five parts of water to one of chloroform to prevent hemorrhage after tooth extraction.

It is not necessary to consider the long list of hæmostatics, as alum, ergot, tannin, etc., but I will say that I do not advise the use of styptic iron or strong solutions of nitrate of silver as hæmostatics in the mouth; in fact, I decidedly object to them both, because of the danger of sloughing and secondary hemorrhage with an enlarged bleeding surface. Nitrate of silver destroys tissue and causes much inflammation to the mucous membrane, and styptic iron makes an objectionable stain which is difficult to eradicate.

For persistent hemorrhage I do recommend, when simpler remedies fail, a sharpened stick of lunar caustic *once* applied as an actual cautery to the bleeding sockets of extracted teeth.

In treating hemorrhage after tooth extraction, my method is this: I have the patient rinse his mouth with an antiseptic solution. I then syringe the bleeding sockets in which the teeth were with pure peroxide of hydrogen or dioxygen, then follow with an antiseptic solution. This stops the hemorrhage, but should it recur I repeat the above, and then apply as an actual cautery a sharpened stick of lunar caustic run down very carefully into the very bottom of each socket, then pack tightly with antiseptic cotton held in place with a compress. The peroxide of hydrogen or dioxygen dissolves more thoroughly the clots from the mouths of the vessels, gives oxygen to the blood, and stimulates the muscular contraction, and nature is allowed to assist itself.

When it is necessary to use an anæsthetic in operating in the oral cavity, chloroform should be chosen (unless contraindicated); as it is not a heart stimulant, it does not increase the blood-pressure, and so adds nothing to the liability to hemorrhage.

During the anæsthesia of the patient, care should be taken not to allow any blood to get into the lungs by way of the trachea, or pneumonia will follow. (Necessary precautions against asphyxiation will of course be taken.) This can be prevented by the Trendelenburg position (from Wm. L. Rodman, A.M., M.D.), the use of the saliva ejector, and judicious sponging with hot water or ice-water.

Of the many operations that are performed in the oral cavity for correcting congenital deformities, for hypertrophy of the gums, for affections of the tongue, the hard and soft palate, extirpation of cysts, tumors, and malignant growths, clipping the uvula, and operations on the tonsils, none are more common than the last named. The following comprehensive definition is given by Dr. Richard Faulkner: "The term 'tonsil' is now applied to various collections of lymphoid glands situated at the oropharyngeal orifice, the anal orifice, and at points throughout the alimentary tract." We have to consider only the oral tonsils. "It is now agreed that they are absorbent glands of lymphoid structure, their chief function being the generation of lymph-cells or leucocytes," which "escape through the surface epithelium of the tonsil into the free cavity of the pharynx, where they destroy micro-organisms and other deleterious agents."

In operating on the tonsils there is usually considerable bleeding, as they are very vascular bodies, deriving their blood-supply from the tonsillar and palatine branches of the facial artery, the descending palatine branch of the internal maxillary, and the ascending pharyngeal. Usually the hemorrhage can be controlled. Fatalities from hemorrhage after amygdalotomy have been caused most often from hæmophilia, or accidental injury to the adjoining larger vessels. Although the internal carotid is situated about three-fourths of an inch back, cases have been recorded where it has been punctured. Brocha reports a fatal case due to an anomaly on this vessel.

Some of our New Orleans fraternity will remember an operation performed here by Dr. Bemis several years ago for abscess of the tonsil. He opened the abscess with a bistoury, and the man went home. After several hours there was a gush of blood. Dr. Bemis was visiting patients and could not be found, and the man bled to death. At the post-mortem examination it was found that the abscess had disintegrated the walls of the carotid artery, and when the pressure exerted by the pus was relieved the walls of the vessel gave way.

The treatment for controlling hemorrhage after extirpation of the tonsils is ligating the larger vessels, where necessary, compression, and styptics. Antipyrin is a favorite hæmostatic in tonsillar surgery.

Dr. Frank Washburn says, "Anæmia is no doubt a causative factor in bleeding after throat operations, as may also be incomplete removal of the adenoids, the wounding of the faucial pillars, reaction after cocaine anæsthesia, etc."

Two cases of severe hemorrhage after the operation of removal of the tonsils are quoted in the last quarterly of the *International Clinics,* one a fatal case, a hæmophilic child, reported by Stewart. I will quote the article exactly for the other case.

Escat reports recovery by operation in a severe case of hemorrhage after amygdalotomy where gargling with cold water, tamponing, using the galvanic cautery, ice, and hæmostatics failed. Record's compressor arrested it, but the pain was so great it had to be removed. The method of Baum was then tried with success. With a needle-holder, used in staphylorrhaphy, he passed a large curved needle and silk thread through the anterior and posterior pillars of the fauces, and tied the suture. This not being sufficient, he tied another two centimetres below. The cavity left by the removal of the tonsil was closed, but the hemorrhage continued. Gelatin was injected into the cavity without result. A cylindrical tampon of cotton roll of the size of his little finger was then carried by a nasopharyngeal forceps from above the upper suture into the cavity and downward until it appeared below the lower suture. The hemorrhage was stopped at the end of twenty-four hours, sutures and tampon removed. The cotton may be previously moistened with adrenalin (1 to 5000), hydrogen peroxide, antipyrin (ten per cent.), or ferropyrin (two per cent.).

